# Revealing the Impact of pH on Lipase Structure and Surface Propensity at the Air–Water Interface and in Aqueous Aerosols

**DOI:** 10.1021/acs.jpclett.5c03315

**Published:** 2026-01-08

**Authors:** Tarun Kumar Roy, Patiemma Rubio, Jenille Cruz, Nicholas A. Wauer, Eshani Hettiarachchi, Rommie E. Amaro, Vicki H. Grassian

**Affiliations:** † Department of Chemistry, 8784 University of California San Diego, La Jolla, California 92093, United States; ‡ Department of Biochemistry and Molecular Biophysics, University of California San Diego, La Jolla, California 92093, United States; § Department of Molecular Biology, University of California San Diego, La Jolla, California 92093, United States

## Abstract

Sea spray aerosols (SSAs), generated through oceanic bubble bursting, are chemically complex particles that significantly influence climate processes and ecosystem health. These aerosols are enriched with biological macromolecules such as enzymes and proteins, whose structure and activity at the air–water interface remain poorly understood, particularly under the highly variable pH conditions of SSAs. In this study, we investigate the pH-dependent surface activity of *Burkholderia cepacia* lipase (BCL), a model extracellular enzyme commonly found in marine environments. Using surface tension and infrared reflection–absorption spectroscopy (IRRAS) measurements, we observe that BCL exhibits increased surface propensity at higher pH compared to acidic conditions. All-atom molecular dynamics simulations further reveal molecular-level insight into these observations, showing structural changes in BCL at the interface in acidic environments with new, highly atmosphere exposed conformations. Additionally, we explore the heterogeneous reactivity of BCL-containing aerosol particles with gaseous nitric acid to identify potential reactive sites relevant to interactions with atmospheric trace gases. Understanding these heterogeneous reaction pathways of biological macromolecules not only may be relevant for SSAs but also has broad implications for the atmospheric reactivity of bioaerosols.

## Graphical abstract

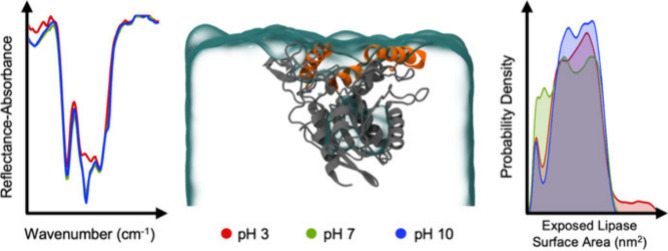

Sea spray aerosols (SSAs), emitted over approximately three-quarters of the Earth’s surface, play a significant role in climate regulation through the formation of cloud condensation nuclei and ice nuclei.^1–6^ SSAs are primarily released into the atmosphere through bubble bursting at the ocean surface and comprise a diverse array of biological macromolecules, including enzymes, proteins, fragmented bacteria, along with organic compounds such as viruses, saccharides, and alkanes.^7–12^ Aerosols containing biological entities, i.e., bioaerosols, are known to impact planetary, human, and ecosystem health.^13–15^ However, the structure and activity of these biomacromolecules at the air–water interface of SSAs remain poorly understood. The air–water interface of an aerosol is critical because it is where biology meets chemistry.^3^ Biological macromolecules within aqueous aerosols can interact with and be influenced by ambient conditions (e.g., temperature and relative humidity) and the presence of other atmospheric gases. This interplay between the aqueous aerosol and the gas-phase that surrounds it ultimately impacts the chemistry and properties of the aerosol.

The chemical environment within SSAs is highly complex.^16^ They exhibit a wide pH range, from highly acidic (pH ∼ 1) to near-neutral or basic (pH ∼ 8), depending on their origin, composition, and environmental conditions.^17–19^ Such variations can impact the protonation states of biological macromolecules, influencing their structure, surface behavior, and catalytic activity. Therefore, investigating the impacts of pH is important to understand the structure, surface propensity, surface dynamics, and chemical reactivity of biological macromolecules in SSA-like environments. Of particular interest to this study, lipases, as well as other microbial enzymes (e.g., protease, alkaline phosphatase, etc.) are found to be transferred from the ocean to the atmosphere upon bubble bursting.^3^ In this work, *Burkholderia cepacia* lipase (BCL), is used as a model enzyme to explore the pH-dependent surface propensity and chemical reactivity of biomacromolecules at the air–water interface and within aqueous microdroplets.

BCL is an extracellular enzyme found in SSAs and is well-characterized in terms of its structure, biology, and enzymatic function.^20–25^ It contains a hydrophobic “lid” region composed of three helices (α4, α5, and α9) that embed at the air–water interface (Figure 1A).^23^ This dynamic “lid” regulates access to the active site of the enzyme, which contains a catalytic triad of residues Ser87, Asp 264, and His286 (shown in cyan in Figure 1A).^26,27^ Titratable residues can be either protonated or deprotonated based on the p*K*_a_ and location of the side chain in a protein. Figure 1B shows the protonation state of BCL’s titratable residues at a given pH (Table S1). The isoelectric point (pI) of this lipase is 5–6.^28,29^ At pH 3, which is below the pI, the titratable residues are more neutrally charged. As the pH increases, the overall charge distribution changes, with the lipase becoming more negatively charged. The most notable difference being that at low pH of 3, most aspartic acid, glutamic acid, and histidine residues are in a protonated state, as expected by their pKas. However, in neutral to basic pH conditions, these same residues can become deprotonated leading to a shift to a more neutrally charged environment among the titratable residues.

**Figure 1:**
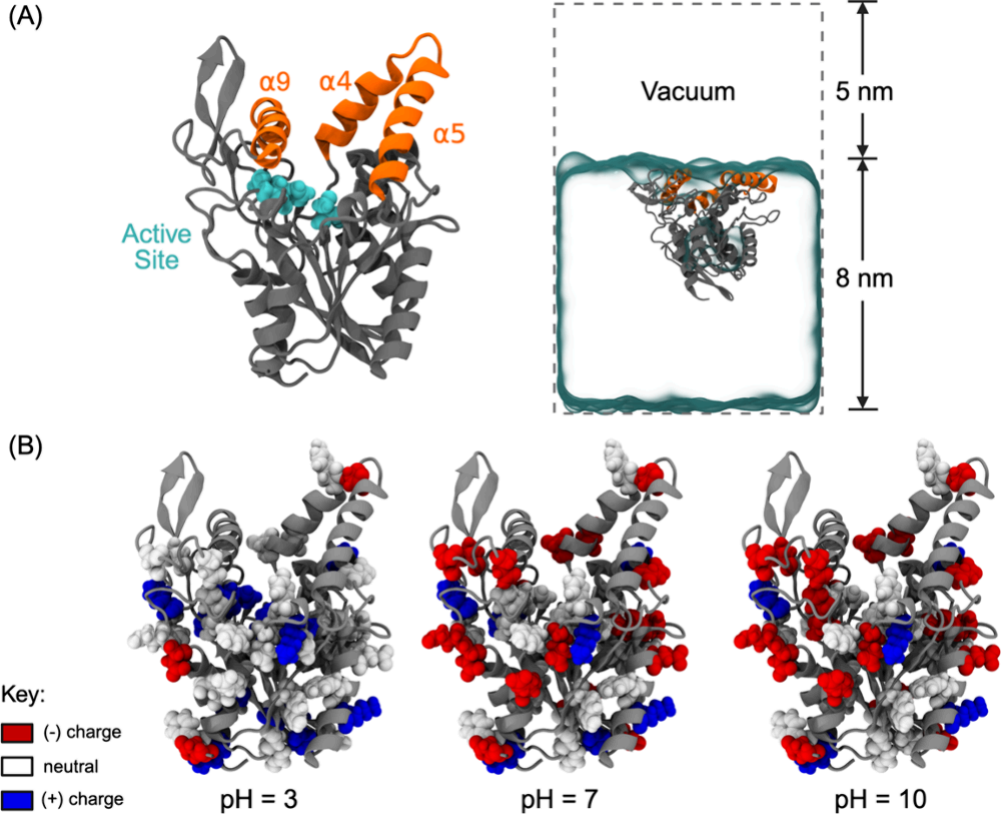
(A) Protein structure of BCL (PDB: 3LIP),^20^ where the hydrophobic lid region is shown in orange and the active site of the protein is shown in cyan (Left). Snapshot of an MD simulation with BCL at the air–water interface, where the lid region of BCL is oriented toward vacuum (atmosphere). Periodic boundary conditions are visualized by the gray dashed box (Right). (B) The charge distribution of titratable residues along the structure of BCL at different pH conditions.

To characterize the interfacial behavior of BCL across a range of pH conditions in sea-like environments, we applied a combination of surface tension measurements and infrared reflection–absorption spectroscopy (IRRAS). All-atom molecular dynamics (MD) simulations were then used to characterize the structure and dynamics of BCL at the air–water interface at different pH conditions, providing molecular-level insights to complement our experimental findings. Both experiments and theory were performed with 0.4 M NaCl solvent to reflect the concentration of seawater and nascent SSAs. Together, this integrated experimental and computational approach advances our understanding of the physicochemical role of enzymes in atmospheric aerosols. This study also explores the heterogeneous reactivity of BCL-containing aerosol particles with atmospheric nitric acid to better understand lipase-mediated biotransformation and its implications for the atmospheric chemistry of bioaerosols.^30^

To investigate how pH influences the surface activity of BCL, we performed surface tension measurements under various BCL concentrations and pH conditions. Figure 2 depicts the surface pressure plotted against BCL concentration in 0.4 M NaCl solutions at pH levels of 3, 7, and 10. Additionally, the surface pressure (π) was determined by subtracting the surface tension of the pure aqueous solution, γ_o_, from that of the BCL-containing solution, γ_BCL,_ using eq [Disp-formula eqE1]:

E1 $$\pi =\gamma_{o}-\gamma_{\mathrm{BCL}}$$

**Figure 2:**
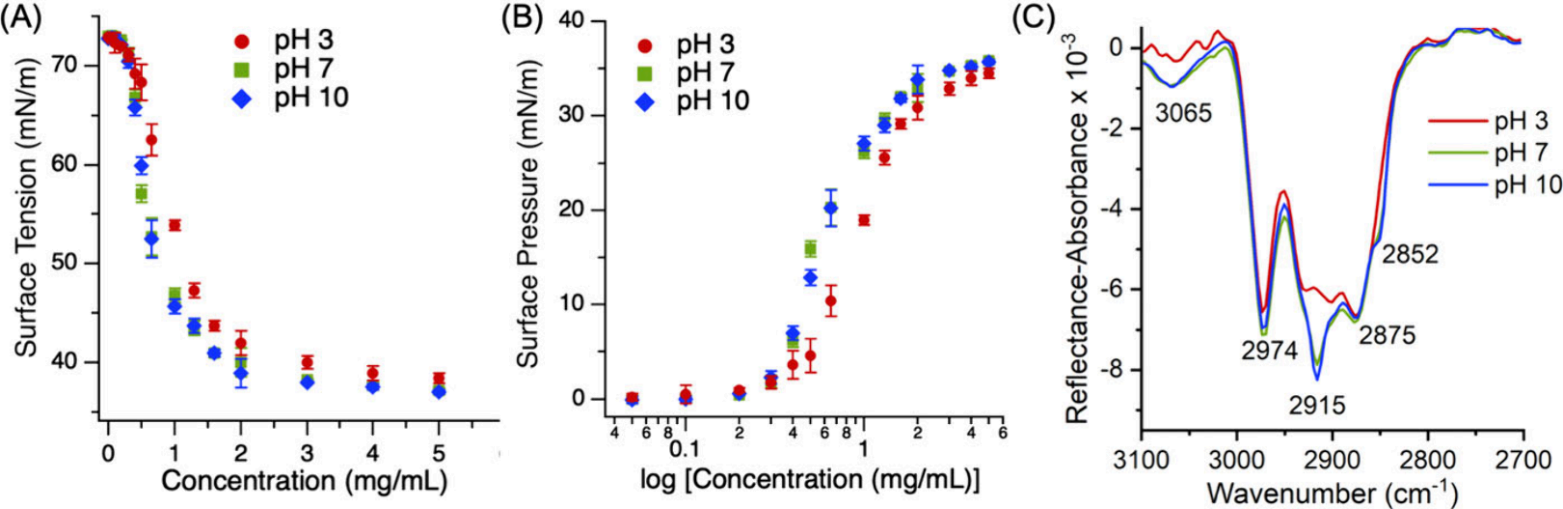
(A) Surface tension of BCL in 0.4 M NaCl solution as a function of concentration at pH 3, 7, and 10. (B) Corresponding surface pressure plotted against BCL concentration on a logarithmic scale, showing lower surface pressure at pH 3 compared to pH 7 and 10. Data points represent the average of three independent measurements, with error bars indicating one standard deviation. (C) Infrared reflection–absorption spectroscopy (IRRAS) spectrum of BCL (1 mg/mL) at the air–water interface in 0.4 M NaCl at different pH levels. Enhanced C–H stretching features are observed at pH 7 and 10 relative to pH 3.

As observed for hydrophobic amino acids, increasing BCL concentration leads to a rise in π, indicating enhanced adsorption of BCL molecules at the interface, which subsequently reduces the surface tension of water. Figure 2B displays a clear increase in surface pressure with increasing BCL concentration, indicating the adsorption of BCL molecules at the air–water interface and the associated reduction in surface tension of water. At low concentrations, the surface pressure remains close to zero, indicating minimal interfacial activity. However, above a critical concentration of 0.4 mg/mL, a sharp rise in surface pressure is observed, corresponding to significant interfacial adsorption of BCL.

The influence of pH on the surface propensity of BCL is evident in the trends shown in Figure 2. The surface pressure profiles for pH 7 and 10 exhibit similar trends, suggesting comparable interfacial behavior in neutral and basic environments. Above 0.4 mg/mL BCL concentration, surface pressure values are consistently lower at pH 3 compared to those at pH 7 and 10, indicating reduced surface activity under acidic conditions. This difference is especially pronounced in the intermediate concentration range of 0.5–2 mg/mL. However, the difference in surface pressure between the pH conditions becomes reduced in the higher concentration regime, suggesting that interfacial saturation of BCL may eventually override the effects of pH.

Figure 2C shows an infrared reflection–absorption spectrum (IRRAS) of BCL at the concentration of 1 mg/mL in 0.4 M NaCl solutions at air–water interfaces at different pH values (3, 7, and 10). The IRRAS spectrum of BCL at the concentration of 2 mg/mL in 0.4 M NaCl solutions is also shown in Figure S1. The spectrum exhibits distinct bands in the C–H stretching region (3100–2700 cm^–1^), corresponding to interfacial functional groups associated with protein side chains. Based on previous sum frequency generation (SFG) spectra of the bovine serum albumin (BSA) protein,^31^ three different functional groups are assigned in this region: methylene, methyl, and phenyl groups. Specifically, the peaks at 2875 cm^–1^ and 2974 cm^–1^ correspond to the symmetric and asymmetric stretches of methyl groups, respectively, while the bands at 2852 cm^–1^ and 2915 cm^–1^ arise from symmetric and asymmetric stretches of methylene groups. The weak shoulder near 3065 cm^–1^ is attributed to the aromatic C–H stretching of phenyl groups. According to the molecular structure of BCL, the methyl group signals originate from side chains of alanine, valine, leucine, isoleucine, and methionine. The aromatic C–H signal stems specifically from phenylalanine, tyrosine, tryptophan, and histidine.

Importantly, it has been demonstrated that IRRAS band intensities do not scale linearly with surface concentration due to factors such as exciton delocalization and the loss of weaker bands in the baseline. The presence of different cations at varying pH can also alter the interfacial water structure, further complicating direct quantitative interpretation. Therefore, we do not attempt to quantify surface enrichment solely from IRRAS intensity. Nevertheless, the increased IRRAS signal intensity (i.e., more negative absorbance) at pH 7 and 10, particularly in the methyl (2974 cm^–1^) and methylene (2915 cm^–1^) regions, suggests greater interfacial packing of BCL under neutral and basic conditions compared to pH 3. These observations are consistent with our surface tension and surface pressure measurements, indicating that BCL exhibits enhanced interfacial adsorption or structural organization at higher pH values. The reduced surface propensity at acidic pH suggests a less compact interfacial arrangement, possibly due to protonation-induced conformational changes. Overall, these observations indicate that pH influences the interfacial conformation and packing of BCL through protonation-dependent structural rearrangements of associated amino acids.

To further explore the effects of varying pH conditions on BCL, all-atom MD simulations were performed to analyze BCL dynamics on the molecular level. More specifically, we examined the structural character of BCL at the air–water interface in our simulations. For all pH environments (3, 7, and 10), as the simulation progressed, the singular BCL protein would move from bulk solution to the air–water interface. With each simulation running at different starting velocities to ensure the creation of independent replicates, some replicates at neutral pH were observed to spend a significant amount of time in the bulk solution before partitioning to the surface ().

Once at the surface, BCL was found to be exposed to the atmosphere above as seen in Figure 3A. The exposure to the atmosphere was quantified across all replicates for each pH condition by determining how much of the lipase’s surface area was not covered by solvent (Figure 3B). These data show that, across the three pH conditions, most lipase conformations have an exposed surface area in the range of 10–30 nm^2^. The high density of BCL conformations in pH 7 exhibiting an exposed surface area of less than 10 nm^2^ can be attributed to the duration of lipase in bulk solution. Interestingly, there are some lipase conformations with an atmosphere-exposed area greater than 40 nm^2^ in simulations for acidic conditions. Although the greater than 40 nm^2^ region is not as densely populous, upon conferring with the per replicate lipase exposure data in Figure S2, lipase becoming largely exposed to the atmosphere above it at the air/water occurred in the last 100 ns of simulation for two of the five total replicates at pH 3. This implies that lipase at pH 3 could become more exposed at the air–water interface if simulations were performed for longer periods of time. The dynamic, hydrophobic lid of BCL was identified to be the predominant region found to be atmosphere-exposed across all pH environments.

**Figure 3:**
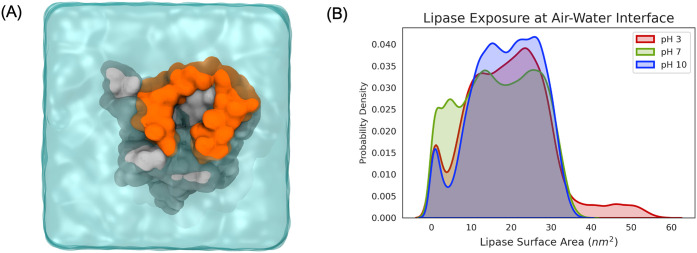
(A) Top-down snapshot of an MD simulation with a protein surface representation of BCL at the air–water interface. This view shows the region of BCL exposed to the atmosphere, where the lid region of the lipase is colored orange. (B) Lipase surface area exposed at the air–water interface, as a distribution of conformation probability density.

BCL lid dynamics were further examined to determine how variations in aerosol acidity influence enzyme structure at the air–water interface. When BCL transitions between open and closed states, this movement is primarily mediated by a displacement of the α5 helix away from or toward the α9 helix. To quantify this interfacial motion of the lipase lid region, the distance between the two helices was calculated across the simulations (Figure 4A and Figure S3). Specifically, the radius of the center of mass of the α carbon atoms was measured between residues 138–142 in α5 and residues 246–251 in α9. These residue selections were guided by prior studies reporting lid distance measurements.^32–34^ The results show that in neutral and basic environments the majority of lipase conformations exhibit a lid distance of approximately 2.4 nm (Figure 4B left and Figure S3). However, for data collected from pH 3 simulations, the distribution shifts toward a larger lid distance. These results suggest an overall more open lid in acidic environments, with the most common lipase conformations measuring a lid distance of ca. 3.0 nm (Figure 4B middle and Figure S3). Similarly to the lipase exposure data, the last 100 ns of simulation is when lipase is most open, compared to the earlier portions of the simulation (Figure S3), and this is observed for several replicates across pH systems. This may suggest that the longer the lipase localizes at the air–water interface, the more open the lid of BCL becomes.

**Figure 4:**
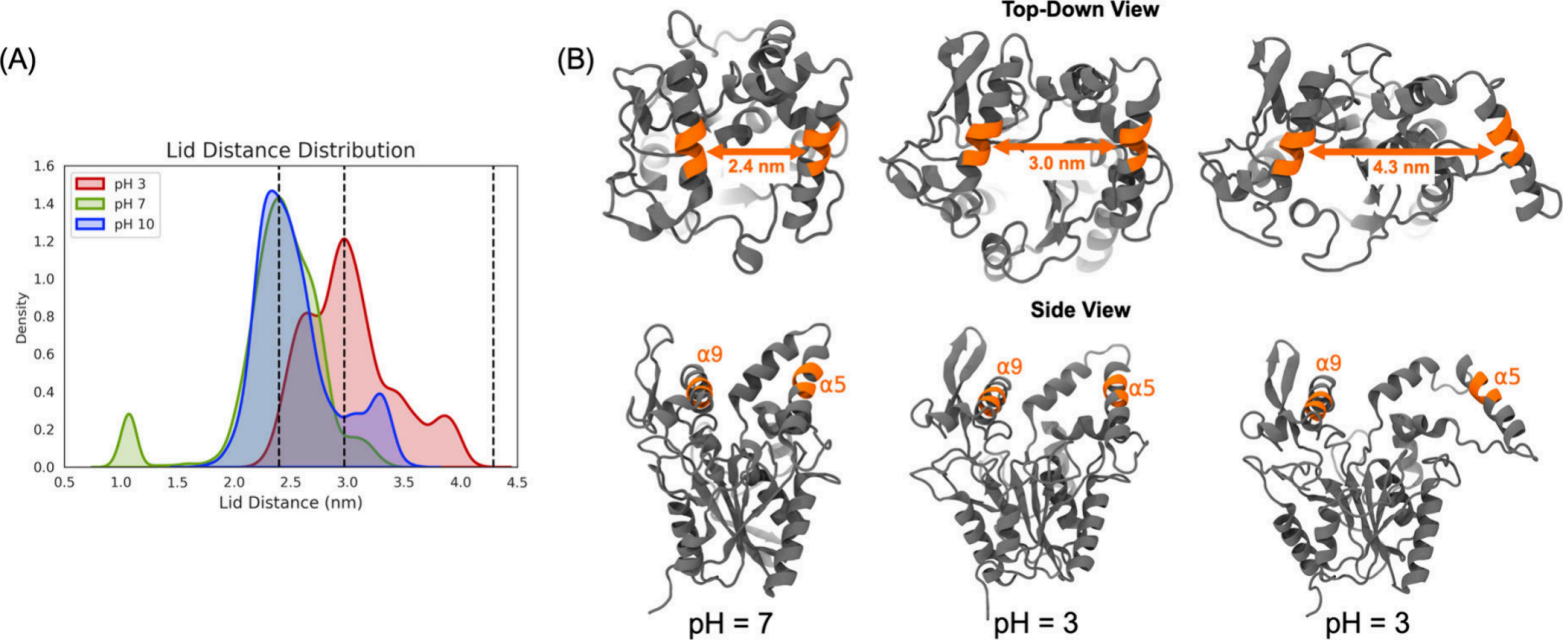
(A) Distribution of BCL lid distance across all simulations performed in different pH environments. From left to right, the vertical dashed lines highlight the lid distance with the most populous protein conformations in pH 7 and pH 3 simulations and the largest lid distance calculated in pH 3 simulations. (B) Top-down and side views of BCL’s protein structure at pH 7 and 3 corresponding to the emphasized distances exhibited in (A). The mode of lid distance conformations at pH 7 is 2.4 nm. The mode of lid distance conformations at pH 3 is 3.0 nm. The most open confirmation observed at pH 3 has a lid distance of approximately 4.3 nm. These lid distances and their corresponding structure are shown in orange. Likewise, residues in orange along the α5 and α9 helices (residues 138–142 and 246–251, respectively) highlight the regions of the protein used for lid distance calculations.

Since the late-time increase in atmospheric lipase exposure and lid opening occurred in two pH 3 replicates, these two simulations were extended to a total of 600 ns of sampling time to determine whether the behavior reflected insufficient sampling. The lid distance and RMSD structural metrics (Figures S7 and S8) remained stable throughout the extended portion of the trajectories, indicating that the protein structure had already equilibrated. The surface-exposed area (Figure S6) continued to fluctuate, as expected for a protein at a dynamic air–water interface. These results support that the late-time increases observed in the original runs represent accessible interfacial configurations rather than incomplete equilibration. Full analyses are provided in the Supporting Information.

Overall, the pH 3 BCL lid structure differs significantly from the other pH values with a greater range of common lid distances. Particularly interesting is the upper limit of the data, revealing a maximum length between α5 and α9 of 4.3 nm (Figure 4B right). At this distance, BCL is observed to be in a very open state along with having a significantly less compact structure. Although this behavior was not observed in all replicates in pH 3 (Figure S3), it is possible that if more replicates were run or, conversely, production steps were run for a longer time frame, there could be a higher prevalence of these “very open state” lipase conformations. When looking closer into the structure of BCL, our simulations show that the α5 helix shifts further away from α9, when an adjacent loop no longer interacts with α5; a behavior not seen at the other pH values. These conformational changes in BCL could imply interfacial instability of lipase at lower pH levels. This reduced structural stability of lipase at the air–water interface may correlate with the reduced surface propensity at acidic levels found experimentally. Recall the key structural differences between pH 3 compared to pH 7 and 10 are the residues Asp, Glu, and His being protonated at pH 3 but deprotonated at the higher pH values, pH 7 and 10. Moreover, demonstrating that changes in protonation state play a fundamental role in the pH-dependent nature of BCL at the air–water interface.

Although these pH-dependent conformational trends suggest that BCL structure and interfacial stability vary across aerosol-relevant conditions, they cannot be directly translated into predictions of catalytic activity. Classical MD simulations, as applied here, cannot capture enzymatic turnover with bond formation or cleavage. Instead, the simulations characterize how protonation-state changes and interfacial localization influence lipase flexibility and lid accessibility. Previous studies found that experimentally, BCL exhibits greater catalytic activity in bulk solution at pH values above 7 and retains substantial activity under basic conditions.^35^ In our simulations, the active-site region remains accessible across all pH environments, and in some cases becomes even more open; notably, the active site never transitions into a closed conformation. While increased lid opening at low pH could hypothetically influence catalytic competency or contribute to reduced stability, such effects cannot be inferred from MD alone and would require targeted biochemical assays that are beyond the scope of this study. Thus, the structural behaviors described here should be interpreted as pH-dependent dynamical responses rather than direct indicators of catalytic activity.

Another aspect of this study is to investigate the heterogeneous reactivity of aerosol particles composed of BCL with acidic trace atmospheric gases. Specifically, here we are investigating the reactivity of nitric acid (HNO_3_) and probing the heterogeneous chemistry with micro-Raman spectroscopy. This approach aims to elucidate the reactivity of bioaerosols, which typically comprise of a diverse array of biological molecules and offer numerous reactive sites for interactions with atmospheric trace gases. Understanding how lipase-mediated biotransformation occurs in SSAs has broad implications for the atmospheric reactivity of bioaerosols.^30^

Figure 5 shows the Raman spectrum of BCL aerosol particles (5–10 μm) deposited on a substrate from an aqueous solution and dried, before and after exposure to nitric acid vapor (20 mTorr) at a relative humidity (RH) of ca. 40%. Upon exposure of BCL particles to HNO_3_, the symmetric stretch (ν_1_) of the nitrate ion became evident at 1048 cm^–1^, indicating the particle has reacted. A comparable nitrate peak was observed in our previous study on the reaction of gaseous HNO_3_ with lipopolysaccharide (LPS) aerosol particles.^36^ These results demonstrate that following reaction, there is dissociation of HNO_3_ to yield H^+^ and NO_3_^–^ due to the protonation of BCL. The nitrate anion shows distinct signal in the Raman spectrum associated with the symmetric stretch at 1048 cm^–1^. These results further show how micro-Raman spectroscopy can be used as a tool for identifying the heterogeneous reactivity of bioaerosol particles.

**Figure 5:**
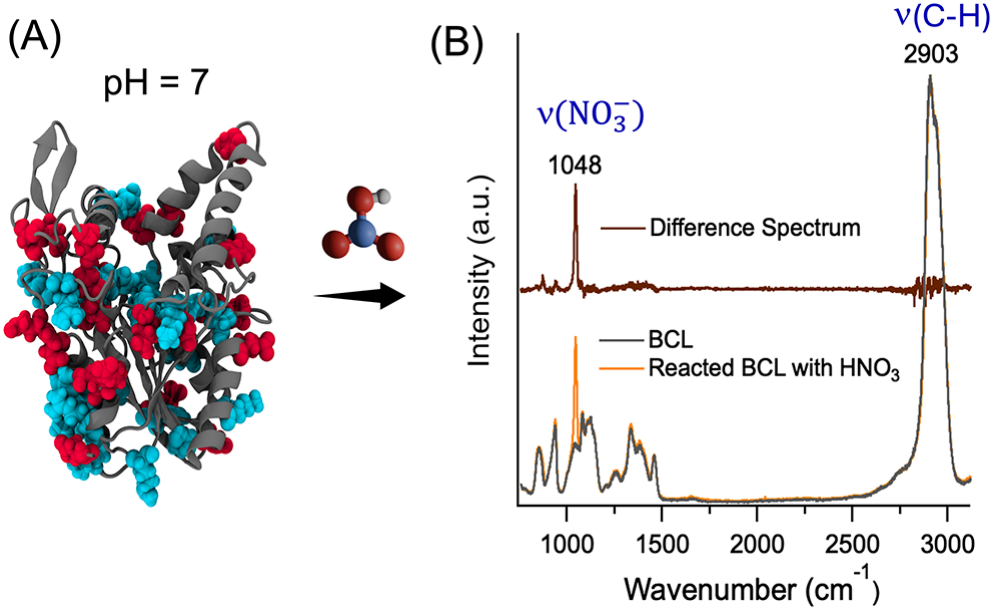
(A) Structure of BCL at pH 7, where the red residues represent the amino acids containing a −COOH side chain (Asp, Glu) and the blue residues represent the amino acids that have titratable nitrogen-containing side chains (Lys, Arg, His). The arrow indicates heterogeneous reaction of BCL containing bioaerosols with gas-phase nitric acid (HNO_3_). (B) Raman spectra were collected for substrate-deposited BCL aerosol particles (5–10 μm) from aqueous solution before (gray) and after (yellow) exposure to HNO_3_. The 1048 cm^–1^ peak corresponds to the ν_1_ symmetric stretch of the nitrate ion, indicating the heterogeneous reactivity of BCL with HNO_3_, can be easily seen in the difference spectrum, after exposure minus before exposure, (red).

The pH of the BCL aqueous solution is ca. 6.2–7, a condition under which acidic components such as HNO_3_ can protonate or interact with its basic and anionic functional groups. Note that the reaction of BCL aerosol particles was carried out for the particles themselves, without sodium chloride present to avoid the well-known reaction between nitric acid gas and the sodium chloride present in SSAs, as shown in Reaction [Disp-formula eqR1].

R1 $$NaCl+HNO_{3}\to NaNO_{3}+HCl$$

Based on the amino acid composition of BCL (Table S2), the protein contains several reactive residues that can contribute to its reactivity toward HNO_3_. Basic amino acids such as lysine (2.2% of total residue count), arginine (2.8%), and histidine (1.9%), which can have protonated or partially protonated nitrogen groups at pH ca. 6.5 that can interact with nitrate anions or undergo further protonation, as shown in Reaction [Disp-formula eqR2]. Additionally, acidic residues like aspartic acid (4.7%) and glutamic acid (2.2%) are likely to be deprotonated at this pH, existing as carboxylates that can engage in proton transfer reaction with nitric acid (Reaction [Disp-formula eqR3]). The presence of these functional groups in BCL provides a molecular basis for the observed formation of nitrate species, as evidenced by the Raman spectrum.

R2 $$BCL\text{−}NH_{2}+HNO_{3}\to BCL\text{−}NH_{3}^{+}+NO_{3}^{-}$$

R3 $$\mathrm{BCL}\text{−}{\mathrm{COO}}^{-}+HNO_{3}\to BCL\text{−}COOH+NO_{3}^{-}$$

In summary, we present an integrated experimental and computational approach to investigate the interfacial behavior of BCL across a range of pH conditions in sea-like environments. Surface tension and IRRAS measurements revealed that BCL exhibits enhanced surface propensity at higher pH values (7 and 10) relative to acidic conditions (pH 3), suggesting increased interfacial adsorption or structural organization in alkaline environments. Complementary MD simulations further reveal that deprotonation of acidic residues at higher pH promotes stronger interfacial localization and more compact packing, whereas at pH 3 the lipase remains more exposed at the air–water interface with an extended open-lid conformation (∼3.0 nm vs ∼2.0 nm lid distance). We also show the heterogeneous reactivity of BCL-containing aerosol particles with gaseous nitric acid and identify potential reactive sites relevant to interactions with trace atmospheric gases. Altogether, our findings highlight the central role of pH-dependent conformational states in governing both interfacial organization and chemical reactivity of bioaerosols, with broad implications for atmospheric processes.

## Supplementary Material











## Notes

SI-1 provides a description of experimental and computational methods. The eight figures included in SI-1 are IRRAS spectra of BCL at different concentrations (Figure S1); BCL surface area exposed at the air–water interface over time (Figure S2); BCL lid distance as a function of time (Figure S3); distribution of BCL lid distance across all simulations performed in different pH environments (Figure S4); root-mean-square deviation (RMSD) calculations as a function of time (Figure S5); BCL surface area exposed at the air–water interface over time for the extended simulations (Figure S6); BCL lid distance as a function of time for the extended simulations (Figure S7); and RMSD calculations as a function of time for the extended simulations (Figure S8). The two tables included in SI-1 are protonation states of titratable residues of BCL at different pH (Table S1) and residue composition of BCL (Table S2) (PDF)
p*K*_a_ data obtained from PROpKa (TXT)
Computational script for energy minimization (PDF)
Computational script for system equilibration (PDF)
Computational script for production (PDF)
