# Supplementary material for: Revealing the Impact of pH on Lipase Structure and Surface Propensity at the Air–Water Interface and in Aqueous Aerosols
Source: J Phys Chem Lett. 2026 Jan 8;17(3):818–24. doi: 10.1021/acs.jpclett.5c03315 (PMC12833840; doi:10.1021/acs.jpclett.5c03315)
Supplement: Supplementary file 1 [file jz5c03315_si_001.pdf]

Supporting Information

Revealing the Impact of pH on Lipase Structure and Surface Propensity at the Air–Water Interface and in Aqueous Aerosols

Authors: Tarun Kumar Roy,[1†] Patiemma Rubio,[2†] Jenille Cruz,[1] Nicholas A. Wauer, [1] Eshani Hettiarachchi,[1] Rommie E. Amaro[1,2,3 *] and Vicki H. Grassian[1*]

Affiliations:

[1]Department of Chemistry, University of California San Diego, La Jolla, CA, United States

[2]Department of Biochemistry and Molecular Biophysics, University of California San Diego, La Jolla, CA, United States

[3]Department of Molecular Biology, University of California San Diego, La Jolla, CA, United States

[†] These authors contributed equally.

*** Corresponding authors:**

Rommie E. Amaro: ramaro@ucsd.edu and Vicki H. Grassian: vhgrassian@ucsd.edu

Contents:

• Experimental Methods

• Computational Methods

• Extended MD Simulations at pH 3

• 8 SI Figures and 2 SI Tables

• SI References

Experimental methods:

Materials and sample preparation: *Burkholderia Cepacia* Amano Lipase (≥ 23 U/mg) was purchased from Sigma-Aldrich. Milli-Q water with an electric resistance of 18.2 MΩ was used for the aqueous subphase. NaCl salt (99.8%) was purchased from Fischer Scientific and were purified by baking at 200 °C overnight to remove organic contaminants. We used one subphase for our experiments, NaCl solution (0.4 M), which was chosen to be near that of seawater concentrations. Lipase solutions of varying concentrations were prepared in the 0.4 M NaCl subphase. Adjustments to pH were made with concentrated Hydrochloric acid (1 N stock solution, Thermo Scientic) or sodium hydroxide (1 N stock solution, Thermo Scientific).

Surface tension measurements: Surface tension measurements were carried out using a Kibron AquaPi tensiometer with Teflon sample cups. The tensiometer was calibrated with milliQ water to 72.8 ± 0.1 mN m^{-1}. For each concentration, three independent solutions were prepared, each containing 7 mL of sample. The surface tension of each solution was measured three times, and the average value for each solution was calculated. The reported surface tension at each concentration corresponds to the overall average of the three solutions. The pH of each solution was measured both before and after surface tension measurements to verify consistency.

Infrared Reflection Absorption Spectroscopy (IRRAS): Details of the IRRAS instrumentation have been reported elsewhere.[1] Briefly, the IR beam from a Bruker Tensor 37 infrared spectrometer was directed onto the aqueous solution surface in a Petri dish via two gold mirrors. At the air–water interface, the optimal incident angle for the unpolarized beam was approximately 30°. The reflected beam was focused with a CaF_2 lens onto a ZnSe window MCT detector (Infrared Associates Inc.). Aqueous solutions were prepared hours in advance, and to further reduce interference from water vapor, long purge times (≥ 20 minutes) were used prior to data collection. A background spectrum with the same pH and salt concentration was collected before the corresponding BCL spectrum. Each IRRAS spectrum was an average of 1000 scans with a spectral resolution of 4 cm^{-1}. Given the weak signal and interference from water vapor in other spectral regions, analysis was focused on the C–H stretching region. We used the intensity of the C–H stretching region as a monitor of increasing and decreasing surface activity of BCL.

Reactivity of BCL particle with gaseous HNO_3: To investigate the heterogeneous reactivity of BCL-containing aerosol particles with gaseous nitric acid, pure BCL particles of diameter 5–10 µm were generated from a nebulizer (OMRON MicroAIR U100) containing BCL in an aqueous solution (with no salts or pH adjustment). The aerosol particle was deposited onto a quartz substrate and dried. The BCL-containing aerosol particles were then exposed to ~20 mTorr of HNO_3 at ~40% RH. Raman measurements were carried out using a confocal Raman spectrometer (HORIBA LabRam HR Evolution) operated with LabSpec 8 software. The instrument was equipped with an Olympus BX41 optical microscope, employing both 10× and 100× super-long working distance objectives, and a 532 nm excitation laser. A confocal Raman spectrometer (HORIBA, LabRam HR Evolution) was used with the LabSpec 8 sotware. Raman spectra of the particles were recorded following 30 minutes of exposure to HNO_3 vapor. The extent of reaction was then determined by summing the total counts in the nitrate ν1 stretching region (1048 cm^{-1}).

Computational methods:

Molecular Dynamics System Preparation: All solvent systems were set up using CHARMM-GUI in 0.4 M NaCl at 298.15 K and were composed of a rectangular box with periodic boundary conditions, utilizing the Solution Builder method.[2-4] The water box was 8 x 8 x 8 nm, the system box was later expanded in the z-direction to 13 nm, incorporating an air (vacuum) interface. A diagram of the system set up is shown in Figure 1A. The TIP3P water model was selected for its compatibility with CHARMM36m[2-5] force field and to be consistent with previously constructed lipase models[6]. The starting lipase structure used for our simulations was based on the 3LIP PDB entry, with protonation states for all residues assigned corresponding to the pH values of interest (3, 7, 10) using PROpKa to determine the relative pKa of each titratable residue (Table S1).[7-9] The pKa data from PROpKa can be found in Supplemental File 2. Each system contained a singular lipase which was placed in the center of the water box. The constructed systems each consisted of approximately 47,000 atoms. All systems were prepared for simulation with GROMACS and parametrized using the CHARMM36m force field. The solvent systems were energy-minimized and equilibrated using GROMACS 2023[10-16] on the San Diego Supercomputer Center (SDSC) Triton Shared Computing Cluster (TSCC) Hopper[17].

Molecular Dynamics Simulations and Data Analysis: The energy minimization and equilibration settings used were unchanged from the default script provided by CHARMM-GUI, found in Supplemental Files 3 and 4. Production steps for each system were run for approximately 300 ns per replication for a total of five replicates, using an NVT ensemble to ensure periodic boundary conditions were maintained conserving the constructed air-water interface. The systems were determined to be fully equilibrated when root-mean-square deviation (RMSD) data converged (Figure S4). All production runs were run using allocations for the Amaro Group using the SDSC TSCC Hopper. The production run script is given in Supplemental File 5. Visual Molecular Dynamics (VMD)[18] was used to visualize and render system simulations. Molecular simulation data was analyzed using a combination of GROMACS[10-16] (gmx) and Python[19] packages. To calculate the atmosphere-exposed lipase area over time, the gmx sasa function in GROMACS calculated the protein surface area contributing to the total atmosphere exposed surface area in the system (Figure 3B, Figure S2 and S6). Likewise for lid distance calculations, an index of residues 138-142 in α5 and residues 246-251 in α9 helices of BCL was created with gmx make_ndx and the distance between these two segments were measured using gmx distance (Figure 4A, Figure S3, S4A, and S7). Determining convergence in simulation data was done using the Python package mdtraj[20] and numpy[21] for calculating RMSD (Figure S5 and S8). Lastly, calculated data was plotted using the matplotlib[22] Python library in the Jupyter Notebook environment.

Extended MD Simulations at pH 3:

To assess whether the late-time atmosphere-exposed surface area observed in two pH 3 replicates reflected insufficient sampling, we extended these trajectories from 300 ns to a total of 600 ns. Atmosphere-exposed lipase surface area, lid distance, and RMSD were calculated for the extended replicates similar to the analyses performed for each pH system.

Consistent with the initial 300-ns results, RMSD values reached a stable regime early in the simulation and remained within a narrow distribution for the remainder of the trajectory (Figure S8). Lid-distance fluctuations likewise stabilized after the early portion of the simulations (Figure

S7), indicating no evidence of structural drift or progressive opening of the lid domain. In contrast, the atmosphere-exposed lipase surface area (Figure S6) continued to fluctuate over a relatively broad range throughout the extended simulations. These fluctuations reflect the dynamic nature of the protein positioning at the air–water interface (Graham, D. E. et al., J. *Colloid Interface Sci.* 1979, 70 (3), 403-414 and Dommer, A. C. et al. *ACS Cent. Sci.* **2023**, *9*(6), 1088–1103.) rather than a lack of structural equilibration. The extended simulations showed that the larger exposure values (>40 nm²) sampled in the original trajectories represent accessible but not progressively increasing states, with the system exhibiting reversible transitions between more and less exposed configurations.

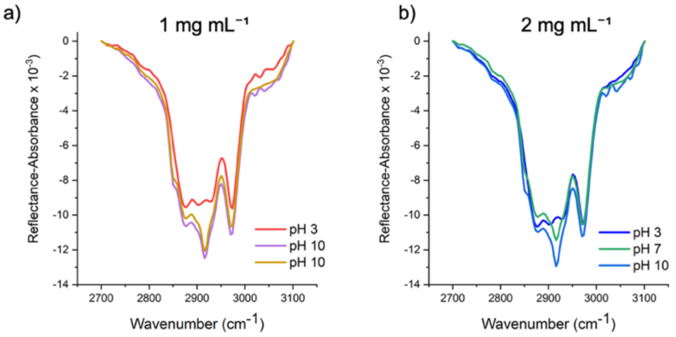

Figure S1. Infrared reflection-absorption spectrum (IRRAS) of BCL at the concentration of 1 mg/mL (a) and 1 mg/mL (b) in 0.4 M NaCl solutions at air−water interfaces at different pH values (3, 7, and 10).

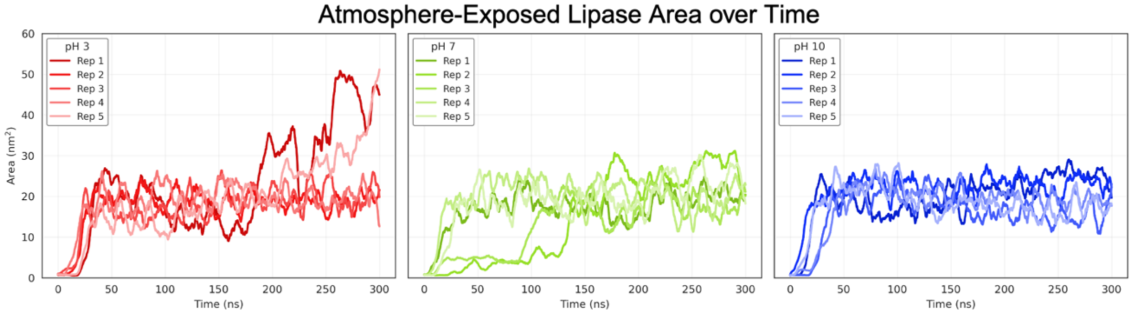

Figure S2. Lipase surface area exposed to the atmosphere at the air-water interface as a function of time for each replicate per pH system.

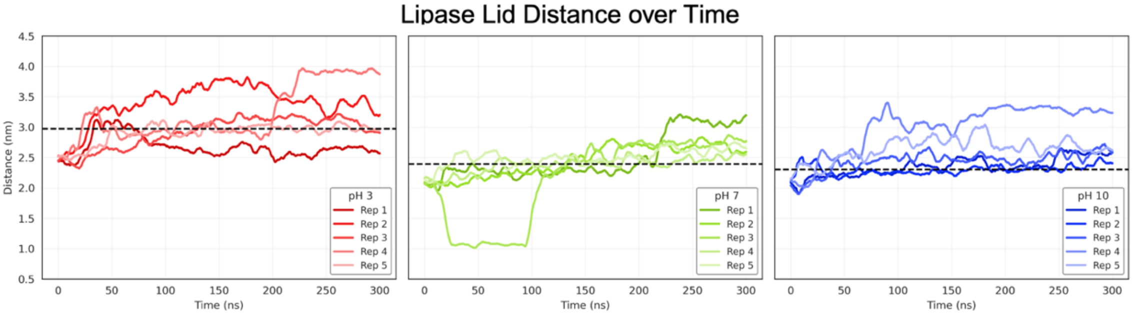

Figure S3. Lipase lid distance as a function of time for each replicate per pH system. The dashed horizontal lines identify the most populous lid distance occupied by the lipase conformations;

similar to that of in Figure 4A and Figure S3. The mode of lid distance conformations are 3.0 nm, 2.4 nm, and 2.3 nm for pH 3, 7, and 10, respectively.

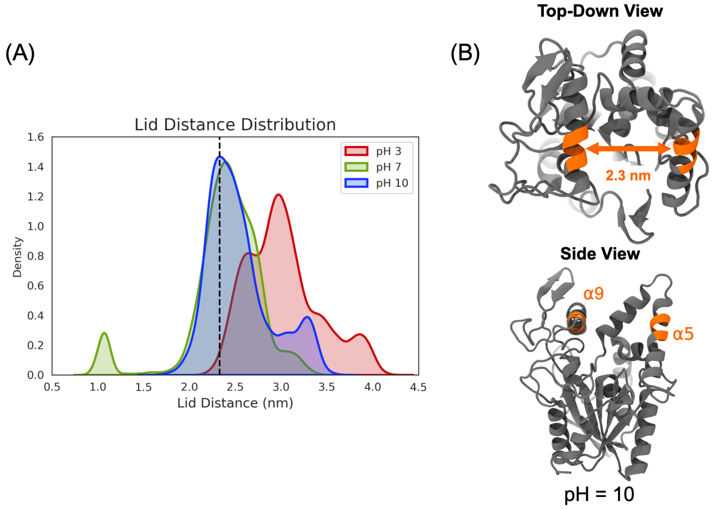

Figure S4. (A) Distribution of BCL lid distance across all simulations performed in different pH environments. The vertical dashed line highlights the lid distance with the most populous protein conformations in pH 10 simulations. (B) Top-down and side view of BCL's protein structure at pH 10 corresponding to the highlight distance exhibited in S2A. The mode of lid distance conformations at pH 10 is 2.3 nm; this lid distance and its corresponding structure are shown in orange. Residues in orange along the α5 and α9 helices (residues 138-142 and 246-251, respectively), highlight the regions of the protein used for lid distance calculations, similar to those in Figure 4.

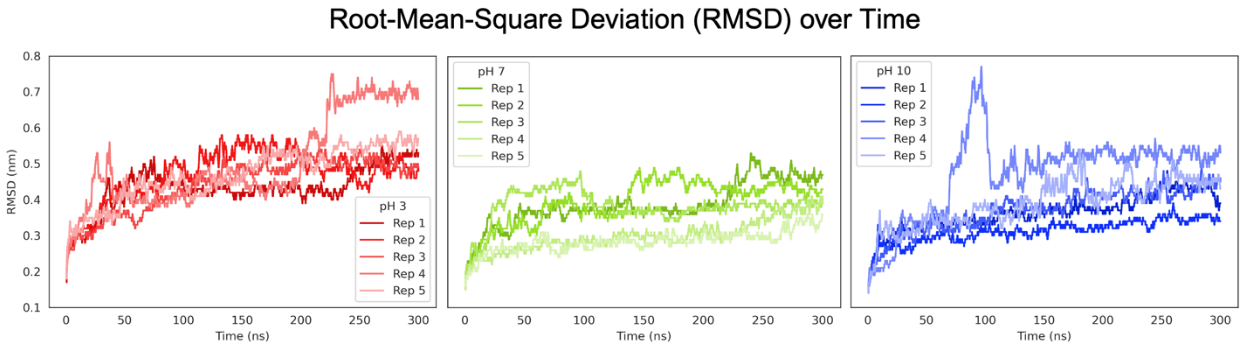

Figure S5. Root-mean-square deviation (RMSD) calculations as a function of time for each replicate per pH system.

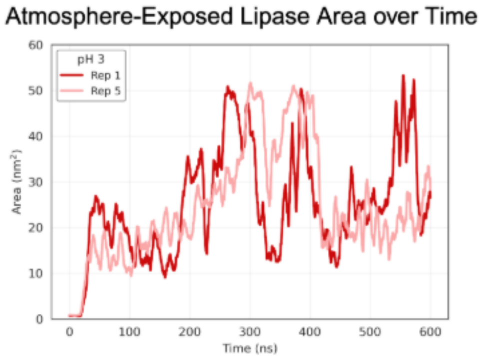

Figure S6. Lipase surface area exposed to the atmosphere at the air-water interface as a function of time for the two pH 3 replicates extended to 600 ns.

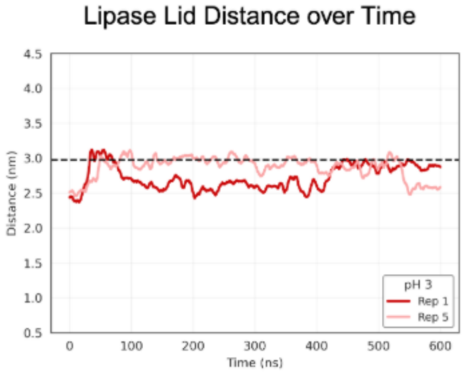

Figure S7. Lipase lid distance as a function of time for the two pH 3 replicates extended to 600 ns. The dashed line indicates the mode of lid distance conformations at pH 3 which is 3.0 nm.

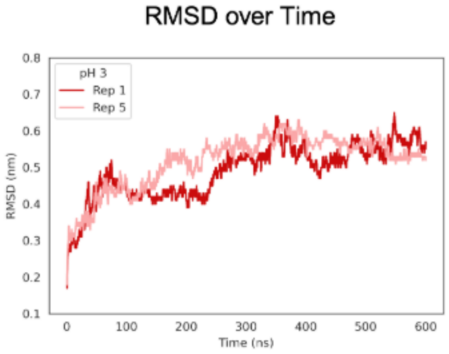

Figure S8. RMSD calculations as a function of time for the two pH 3 replicates extended to 600 ns.

Table S1. Protonation states of titratable residues of BCL at different pH.[8]

Residue	Residue Number	pH 3	pH 7	pH 10
ASP	2, 130, 303	deprotonated	deprotonated	deprotonated
	21, 36, 55, 56, 102, 121, 159, 228, 236, 242, 264	protonated	deprotonated	deprotonated
	288	protonated	protonated	deprotonated
GLU	28, 35, 63, 118, 197, 302	protonated	deprotonated	deprotonated
	289	protonated	protonated	deprotonated
HIS	15, 86, 114, 204, 286, 311	protonated	deprotonated	deprotonated
TYR	4, 9, 23, 29, 31, 45, 68, 95, 129, 175, 179, 207, 274, 282	protonated	protonated	protonated
LYS	22, 70, 165, 283, 316	protonated	protonated	protonated
	80, 269	protonated	protonated	deprotonated
ARG	22, 70, 80, 165, 269, 283, 316	protonated	protonated	protonated

Table S2. Residue composition of BCL.[8]

Residue	Composition Percentage*	Residue Number
ALA	12.2	1, 5, 6, 24, 42, 47, 67, 74, 75, 78, 97, 98, 100, 105, 120, 128, 140, 141, 160, 162, 163, 170, 172, 173, 182, 186, 194, 210, 213, 226, 238, 240, 247, 272, 299, 301, 306, 312, 318
CYS	0.6	190, 270
ASP	4.7	2, 21, 36, 55, 56, 102, 121, 130, 159, 228, 236, 242, 264, 288, 303
GLU	2.2	28, 35, 63, 118, 197, 289, 302
PHE	2.2	52, 119, 122, 142, 146, 221, 249
GLY	11.3	16, 19, 25, 32, 41, 51, 57, 60, 62, 77, 85, 89, 90, 111, 116, 125, 133, 147, 183, 185, 188, 193, 200, 201, 211, 222, 225, 250, 252, 259, 261, 265, 275, 295, 298, 319
HIS	1.9	15, 86, 114, 204, 286, 311
ILE	3.4	11, 12, 33, 110, 139, 148, 214, 218, 232, 290, 308
LYS	2.2	22, 70, 80, 165, 269, 283, 316
LEU	9.7	13, 17, 27, 37, 49, 65, 66, 73, 83, 91, 103, 127, 134, 149, 161, 164, 167, 184, 205, 206, 234, 241, 246, 248, 273, 278, 287, 293, 294, 315, 317
MET	0.3	255
ASN	5.6	3, 48, 59, 82, 144, 154, 155, 157, 176, 178, 202, 239, 257, 263, 285, 291, 300, 313
PRO	4.1	10, 58, 101, 113, 131, 180, 187, 195, 216, 233, 237, 243, 304
GLN	4.7	34, 38, 39, 53, 64, 88, 124, 158, 171, 177, 191, 215, 262, 276, 292
ARG	2.8	8, 40, 61, 94, 115, 258, 297, 309, 314

SER	6.9	50, 54, 87, 93, 106, 117, 135, 136, 151, 152, 153, 181, 189, 208, 219, 230, 244, 260, 268, 271, 279, 281
THR	10.6	7, 18, 20, 43, 71, 76, 79, 92, 108, 109, 112, 132, 137, 150, 156, 166, 168, 169, 174, 192, 196, 198, 203, 212, 217, 224, 227, 229, 231, 245, 251, 253, 280, 310
VAL	9.4	14, 26, 44, 46, 69, 72, 81, 84, 96, 99, 104, 107, 123, 126, 138, 143, 145, 199, 220, 223, 235, 254, 256, 266, 267, 277, 296, 305, 307, 320
TRP	0.9	30, 209, 284
TYR	4.4	4, 9, 23, 29, 31, 45, 68, 95, 129, 175, 179, 207, 274, 282

* Percentage determined by the number of residues per amino acid type compared to the total number of residues found in BCL.

References:

(1) Shrestha, M.; Luo, M.; Li, Y.; Xiang, B.; Xiong, W.; Grassian, V. H. Let there be light: stability of palmitic acid monolayers at the air/salt water interface in the presence and absence of simulated solar light and a photosensitizer. *Chem. Sci.,* **2018**, *9* (26), 5716-5723.
(2) Jo, S.; Kim, T.; Iyer, V. G.; Im, W. CHARMM-GUI: A web-based graphical user interface for CHARMM. *J. Comput. Chem.* **2008**, *29* (11), 1859-1865.
(3) Brooks, B. R.; Brooks Iii, C. L.; Mackerell Jr, A. D.; Nilsson, L.; Petrella, R. J.; Roux, B.; Won, Y.; Archontis, G.; Bartels, C.; Boresch, S.; et al. CHARMM: The biomolecular simulation program. *J. Comput. Chem.* **2009**, *30* (10), 1545-1614.
(4) Lee, J.; Cheng, X.; Swails, J. M.; Yeom, M. S.; Eastman, P. K.; Lemkul, J. A.; Wei, S.; Buckner, J.; Jeong, J. C.; Qi, Y.; et al. CHARMM-GUI Input Generator for NAMD, GROMACS, AMBER, OpenMM, and CHARMM/OpenMM Simulations Using the CHARMM36 Additive Force Field. *J. Chem. Theory Comput.* **2016**, *12* (1), 405-413.
(5) Huang, J.; Rauscher, S.; Nawrocki, G.; Ran, T.; Feig, M.; de Groot, B. L.; Grubmüller, H.; MacKerell, A. D. CHARMM36m: an improved force field for folded and intrinsically disordered proteins. *Nat. Methods* **2017**, *14* (1), 71-73.
(6) Dommer, A. C.; Wauer, N. A.; Angle, K. J.; Davasam, A.; Rubio, P.; Luo, M.; Morris, C. K.; Prather, K. A.; Grassian, V. H.; Amaro, R. E. Revealing the Impacts of Chemical Complexity on Submicrometer Sea Spray Aerosol Morphology. *ACS Cent. Sci.* **2023**, *9* (6), 1088-1103.
(7) Søndergaard, C. R.; Olsson, M. H. M.; Rostkowski, M.; Jensen, J. H. Improved Treatment of Ligands and Coupling Effects in Empirical Calculation and Rationalization of pKa Values. *J. Chem. Theory Comput.* **2011**, *7* (7), 2284-2295.
(8) Schrag, J. D.; Li, Y.; Cygler, M.; Lang, D.; Burgdorf, T.; Hecht, H.-J.; Schmid, R.; Schomburg, D.; Rydel, T. J.; Oliver, J. D.; et al. The open conformation of a Pseudomonas lipase. *Structure* **1997**, *5* (2), 187-202.
(9) Olsson, M. H. M.; Søndergaard, C. R.; Rostkowski, M.; Jensen, J. H. PROPKA3: Consistent Treatment of Internal and Surface Residues in Empirical pKa Predictions. *J. Chem. Theory Comput.* **2011**, *7* (2), 525-537.
(10) Bekker, H.; Berendsen, H.; Dijkstra, E.; Achterop, S.; Vondrumen, R.; Vanderspoel, D.; Sijbers, A.; Keegstra, H.; Renardus, M. Gromacs-a parallel computer for molecular-dynamics simulations. In *4th international conference on computational physics (PC 92)*, 1993; World Scientific Publishing: pp 252-256.
(11) Berendsen, H. J. C.; van der Spoel, D.; van Drunen, R. GROMACS: A message-passing parallel molecular dynamics implementation. *Comput. Phys. Commun.* **1995**, *91* (1), 43-56.
(12) Lindahl, E.; Hess, B.; van der Spoel, D. GROMACS 3.0: a package for molecular simulation and trajectory analysis. *J. Mol. Model.* **2001**, *7* (8), 306-317.
(13) Van Der Spoel, D.; Lindahl, E.; Hess, B.; Groenhof, G.; Mark, A. E.; Berendsen, H. J. C. GROMACS: Fast, flexible, and free. *J. Comput. Chem.* **2005**, *26* (16), 1701-1718.
(14) Hess, B.; Kutzner, C.; van der Spoel, D.; Lindahl, E. GROMACS 4: Algorithms for Highly Efficient, Load-Balanced, and Scalable Molecular Simulation. *J. Chem. Theory Comput.* **2008**, *4* (3), 435-447.
(15) Pronk, S.; Páll, S.; Schulz, R.; Larsson, P.; Bjelkmar, P.; Apostolov, R.; Shirts, M. R.; Smith, J. C.; Kasson, P. M.; van der Spoel, D.; et al. GROMACS 4.5: a high-throughput and highly parallel open source molecular simulation toolkit. *Bioinformatics* **2013**, *29* (7), 845-854.
(16) Abraham, M. J.; Murtola, T.; Schulz, R.; Páll, S.; Smith, J. C.; Hess, B.; Lindahl, E. GROMACS: High performance molecular simulations through multi-level parallelism from laptops to supercomputers. *SoftwareX* **2015**, *1-2*, 19-25.
(17) San Diego Supercomputer Center (2022): Triton Shared Computing Cluster. University of California, San Diego. Service. https://doi.org/10.57873/T34W2R.
(18) Humphrey, W.; Dalke, A.; Schulten, K. VMD: Visual molecular dynamics. *J. Mole. Graph* **1996**, *14* (1), 33-38.

(19) Van Rossum, G. The Python Library Reference, release 3.8.2. Python Software Foundation. **2020**,
(20) McGibbon, Robert T.; Beauchamp, Kyle A.; Harrigan, Matthew P.; Klein, C.; Swails, Jason M.; Hernández, Carlos X.; Schwantes, Christian R.; Wang, L.-P.; Lane, Thomas J.; Pande, Vijay S. MDTraj: A Modern Open Library for the Analysis of Molecular Dynamics Trajectories. *Biophys. J* **2015**, *109* (8), 1528-1532.
(21) Harris, C. R.; Millman, K. J.; van der Walt, S. J.; Gommers, R.; Virtanen, P.; Cournapeau, D.; Wieser, E.; Taylor, J.; Berg, S.; Smith, N. J.; et al. Array programming with NumPy. *Nature* **2020**, *585* (7825), 357-362.
(22) Hunter, J. D. Matplotlib: A 2D Graphics Environment. *Comput. Sci. Eng.* **2007**, *9* (3), 90-95.
