# Supplementary material for: Revealing the Impact of pH on Lipase Structure and Surface Propensity at the Air–Water Interface and in Aqueous Aerosols
Source: J Phys Chem Lett. 2026 Jan 8;17(3):818–24. doi: 10.1021/acs.jpclett.5c03315 (PMC12833840; doi:10.1021/acs.jpclett.5c03315)
Supplement: Supplementary file 3 [file jz5c03315_si_003.pdf]

```
define                  = -DPOSRES -DPOSRES_FC_BB=400.0
-DPOSRES_FC_SC=40.0
integrator              = steep
emtol                   = 1000.0
nsteps                  = 5000
nstlist                 = 10
cutoff-scheme           = Verlet
rlist                   = 1.2
vdwtype                 = Cut-off
vdw-modifier            = Force-switch
rvdw_switch             = 1.0
rvdw                    = 1.2
coulombtype             = PME
rcoulomb                = 1.2
;
constraints             = h-bonds
constraint_algorithm    = LINCS
```
