# Supplementary material for: Revealing the Impact of pH on Lipase Structure and Surface Propensity at the Air–Water Interface and in Aqueous Aerosols
Source: J Phys Chem Lett. 2026 Jan 8;17(3):818–24. doi: 10.1021/acs.jpclett.5c03315 (PMC12833840; doi:10.1021/acs.jpclett.5c03315)
Supplement: Supplementary file 5 [file jz5c03315_si_005.pdf]

```
integrator              = md
dt                      = 0.002
nsteps                  = 150000000
nstxout-compressed      = 25000
nstxout                 = 25000
nstvout                 = 25000
nstfout                 = 25000
nstcalcenergy           = 100
nstenergy               = 1000
nstlog                  = 1000
;
cutoff-scheme           = Verlet
nstlist                 = 20
vdwtype                 = Cut-off
vdw-modifier            = Force-switch
rvdw_switch             = 1.0
rvdw                    = 1.2
rlist                   = 1.2
rcoulomb                = 1.2
coulombtype             = PME
;
tcoupl                  = v-rescale
tc_grps                 = SOLU SOLV
tau_t                   = 1.0 1.0
ref_t                   = 298.15 298.15
;
;pcoupl                  = C-rescale
;pcoupltype              = isotropic
tau_p                   = 5.0
compressibility         = 4.5e-5
ref_p                   = 1.0
;
constraints             = h-bonds
constraint_algorithm    = LINCS
continuation            = no
gen-vel                 = yes
gen-temp                = 298.15
gen-seed                = -1
;
nstcomm                 = 100
comm_mode               = linear
comm_grps               = SOLU SOLV
;
```
